# RIG-I and MDA5 are modulated by bone morphogenetic protein (BMP6) and are essential for restricting Zika virus infection in human Sertoli cells

**DOI:** 10.3389/fmicb.2022.1062499

**Published:** 2023-01-12

**Authors:** Boonyanudh Jiyarom, Stefanos Giannakopoulos, Daniel P. Strange, Nataliya Panova, Michael Gale, Saguna Verma

**Affiliations:** ^1^Department of Tropical Medicine, Medical Microbiology, and Pharmacology, John A. Burns School of Medicine, University of Hawai’i at Mānoa, Honolulu, HI, United States; ^2^Department of Cell and Molecular Biology, John A. Burns School of Medicine, University of Hawai’i at Mānoa, Honolulu, HI, United States; ^3^John A. Burns School of Medicine, University of Hawai’i at Mānoa, Honolulu, HI, United States; ^4^Department of Immunology, Center for Innate Immunity and Immune Disease, University of Washington School of Medicine, Seattle, WA, United States

**Keywords:** Zika virus, innate immunity, Sertoli cells, testis antiviral immunity, RIG-I, MDA5, BMP6

## Abstract

Sexual transmission of Zika virus (ZIKV) is associated with virus persistence in the testes and shedding in the seminal fluid for months after recovery. We previously demonstrated that ZIKV can establish long-term replication without causing cytotoxicity in human Sertoli cells (SC), responsible for maintaining the immune privileged compartment of seminiferous tubules. Functional gene expression analyses also predicted activation of multiple virus sensing pathways including TLR3, RIG-I, and MDA5. Here, we elucidated which of the RNA virus sensing receptors play a decisive role in restricting ZIKV replication. We show that both poly I:C and IFN-β treatment induced a robust antiviral state and reduced ZIKV replication significantly, suggesting that virus sensing and antiviral signaling are functional in SC. Silencing of TLR3, 7, and 9 did not affect virus replication kinetics; however, both RIG-I and MDA5 played a synergistic role in inducing an anti-ZIKV response. Further, the impact of SC-specific immunosuppressive pathways that collectively regulate SC function, specifically the TGF-β superfamily members, TGF-β, Activin A, and BMP6, on ZIKV replication was investigated. While ZIKV did not modulate the expression of TGF-β and Activin A, BMP6 signaling was suppressed at later stages of infection. Notably, treatment with BMP6 increased IFN-β, p-IRF3, and p-STAT1 levels, and expression of key interferon-stimulated genes including MDA5, suggesting that BMP6 enhances antiviral response in SC. Collectively, this study further delineates the key role of the RIG-I-like receptors in sensing ZIKV in SC, and reveals a novel role of BMP6 in modulating innate immune and antiviral response in the testes.

## Introduction

During the unprecedented 2015–2016 Zika virus (ZIKV) epidemic in the Americas, ZIKV emerged as a teratogenic and sexually transmissible virus, an unexpected finding not reported for other closely related flaviviruses (Lazear and Diamond, 2016; Sun, 2016; Wang and Ling, 2016). ZIKV can establish long-term persistence in the testes without any symptoms and can be detected in the semen for months after viremia has cleared in a large percent of ZIKV serum-positive males (Lazear and Diamond, 2016; Mead et al., 2018; Stassen et al., 2018). Moreover, at least one confirmed case of microcephaly has been directly linked to the sexual transmission of ZIKV (Yarrington et al., 2019), thus establishing a potential link between sexual transmission and fetal abnormalities. In many cases, the viral load was found to be significantly higher (10^5^-fold) in semen than in blood or urine, suggesting that local tissue factors can increase the production of virus in the male reproductive tract (Joguet et al., 2017; Stassen et al., 2018). The presence of ZIKV in both the cell-free fraction of seminal fluid and in the sperm head (Mansuy et al., 2016; Stassen et al., 2018) implicates the immune privileged compartment of the testes as one of the reservoirs of long-term persistence. Although the cellular targets of this virus in the human testis are now well defined, the mechanisms by which ZIKV establishes persistence in testicular cells remain to be fully understood.

Different testicular resident cells including testosterone-producing Leydig cells (LC), testicular macrophages, and Sertoli cells (SC) collectively contribute to an immunosuppressive environment that is critical for maintaining homeostasis and protecting the viability of auto-antigenic germ cells produced during spermatogenesis (Cheng and Mruk, 2012; Kaur et al., 2014). The key immune molecules/pathways critical to maintaining this testicular and seminal physiology include transforming growth factor β (TGF-β) superfamily members like TGF-β1, activin, inhibin, and Bone Morphogenetic Proteins (BMPs; Ni et al., 2019). In the testis, TGF-β family members are highly expressed in SC and regulate multiple physiological functions such as spermatogenesis and steroidogenesis (Ni et al., 2019). BMP6 and activins control SC differentiation *via* the SMAD transcription factors and are vital for maintaining spermatogenesis and immune homeostasis (Wang et al., 2017). The SC are also the primary responder to invading pathogens in the testis (Zhao et al., 2014), however, the immune defense pathways including virus sensing pathways, particularly against RNA viruses, are not well defined at the molecular level. Furthermore, the functional significance of TGF-β superfamily molecules in antiviral immunity is not well defined.

Host pattern recognition receptors (PRRs) including TLRs and RLRs sense the viral dsRNA and activate downstream signaling that leads to activation of TBK1 followed by nuclear translocation of phosphorylated IRF3 and type I IFN production. These IFNs induce several interferon-stimulated genes (ISGs) including MxA, IFIT1, and PRRs like RIG-I and TLR3 that collectively generate an antiviral state in neighboring uninfected cells and block viral replication in infected cells (Schoggins and Rice, 2011). We recently demonstrated that ZIKV can infect different testicular cells except for LC; however, virus replication appears to be most robust in SC compared to spermatogonia stem cells (SSC) and peritubular myoid cells (Strange et al., 2018b, 2019). Profiling of ZIKV-specific response in the SC at the transcriptomics and proteomics level revealed that peak virus replication was associated with induction of TLRs and RLRs, and the type I IFN signaling pathway (Strange et al., 2018a, 2021). We also demonstrated that MxA and IFIT1 proteins are the main ISGs induced by ZIKV in SC and they function as antiviral effectors against ZIKV in this cell type (Strange et al., 2021). Despite the induction of key antiviral pathways, we and others noted that SC support high levels of virus replication for long periods of time (up to 3 weeks post-infection) without significantly affecting the cell viability (Siemann et al., 2017; Kumar et al., 2018; Strange et al., 2018a). Further, ZIKV did not induce a robust inflammatory response (Strange et al., 2018a), which may be one of the reasons why infection is not associated with severe cell death of SC unlike other susceptible cells like human neuronal progenitor cells (Ferraris et al., 2019) and retinal epithelial and endothelial cells (Singh et al., 2017). Although our previous studies indicated that multiple PRR pathways can be activated in response to ZIKV infection, it is not clear which of these pathways play a decisive role in sensing ZIKV and restricting virus spread in SC. Here, we investigated the potential role of different PRR pathways in sensing ZIKV infection in SCs. We also investigated the effect of ZIKV infection on the expression of TGF-β superfamily members and their role in virus outcome and crosstalk with type I IFN signaling.

## Materials and methods

### Cells and virus

Low-passage primary human SC and A549 lung epithelial cells were cultured in DMEM/F-12 and DMEM media, respectively, as described previously (Chusri et al., 2016; Stone et al., 2019; Strange et al., 2021). ZIKV strain PRVABC59 (Human/2015/Puerto Rico) acquired from American Type Culture Collection was propagated once in Vero E6 cells.

### ZIKV infection and recombinant protein treatment

SC and A549 cells cultured in 6-, 24-or 96-well plates were infected with ZIKV MOI of 1 for 1 h at 37°C as described previously (Siemann et al., 2017; Strange et al., 2021). In selected experiments, cells were treated with different recombinant proteins or dsRNA mimic poly I:C (InvivoGen) 24 h before infection. Recombinant human BMP6 (PeproTech) was used at 60 or 100 ng/mL concentration according to the instruction sheet, and 0.1% BSA was used as a control. Recombinant TGF-β1 and Activin A proteins were purchased from the R&D System. TGF-β1 was used at 10 or 15 ng/mL concentration while Activin A was used at 50 ng/mL or 100 ng/mL concentration with the respective vehicle as control. For activation of the innate immune pathways, poly I:C (InvivoGen) was used at the concentration of 10 μg/mL, and human recombinant IFN-β (R&D System) was used at 1 IU or 10 IU. After infection, the cells were replenished with fresh media containing different recombinant proteins or poly I:C in both infected and mock control cells every 24 h. Cells were harvested at 48 or 120 hpi.

### RNA interference

SC grown to 60–70% confluency in a medium without penicillin–streptomycin were then transfected with 30 pmol of small interfering RNAs (siRNAs) using the Lipofectamine RNAiMax kit (Life Technologies). In brief, the siRNA-Lipofectamine RNAiMAX complexes (siRNA in 50 μl of Opti-MEM medium mixed with 3 μl of Lipofectamine RNAiMAX) were added to each well and mixed gently. After 24 h of incubation, half of the media was removed and replenished with fresh media. The silencing efficiency was quantified by RT-PCR and/or Western blotting at 48 hpt. The different pools of siRNAs (Silencer Select, Thermo Fisher) include siRNA for Toll-like receptor 3 (TLR3) (ID: s236), TLR7 (s27844), TLR9 (s28872), RIG-I (ID: s24143 and ID: s223616), and MDA5 (ID: s34498). A nontargeting siRNA was used as a negative control in every experiment (cat: 4404020).

### ZIKV quantitation

ZIKV titers in cell culture supernatants at different time points after infection were analyzed by plaque assay using Vero cells and expressed as ZIKV PFU per mL of supernatant (Siemann et al., 2017). Intracellular viral RNA was extracted from cell lysates at 48 and 120 hpi and was measured by qRT-PCR using primer and probe specific for ZIKV Env region and expressed as PFU equivalents per μg of RNA as described previously (Siemann et al., 2017).

### Analysis of host response

Changes in mRNA transcripts of key antiviral genes were measured from extracted RNA by qRT-PCR, as described previously (Strange et al., 2021). The reference gene *GAPDH* was used to normalize the data and calculate the fold-change of antiviral genes compared to respective mock-infected cells. Specific primer sequences used for *GAPDH, IFNB1*, *IFIT1*, *MXA*, *STAT1, IFIH1 (MDA5)*, and *DDX58 (RIG-I)* transcript amplification have been previously described (Strange et al., 2018a). Forward and reverse primer sequences used for TGF-β family members, *STAT1* and *IRF7* are shown in Table 1.

**Table 1:** Primer sequences used for qRT-PCR.

Gene	Gene bank accession no.	Primer direction	Primer sequence (5′-3′)
*BMP6*	NM_001718.6	Forward	CAGTGCTTCAGATTACAACA
		Reverse	GGTCTTGGAAACTCACATAC
*INHBA*	XM_017012175.1	Forward	AAGGAGGGCAGAAATGAATGA
		Reverse	TCCTGGCTGTTCCTGACT
*TGFB*	NM_000660.7	Forward	CCACAGATCCCCTATTCA
		Reverse	CTCAGTATCCCACGGAAA
*IRF7*	NM_001572.5	Forward	CCTGGTTGGTTTACACAA
		Reverse	CGTGATCCTCTTCAATATCC
*STAT1*	NM_007315	Forward	CAGAGAACAGCAGACCAA
		Reverse	GGGCATTCTGGGTAAGTT

### Western blot

Cell lysates were prepared using M-PER Mammalian Protein Extraction Reagent with a cocktail of protease and phosphatase inhibitors (Cell Signaling). The protein samples were separated on an NU-PAGE Bolt Bis-Tris Plus Gels (Invitrogen), transferred onto nitrocellulose membranes (Bio-Rad), blocked using Intercept (TBS) Blocking Buffer (Li-Cor Biosciences), and then incubated overnight with different antibodies (Strange et al., 2019). Specific primary monoclonal antibodies used were rabbit anti-human against RIG-I (Cell Signaling Cat. No: 3743S), MDA5 (Invitrogen Cat. No: 700360), MXA (Santa Cruz Biotechnology Cat. No: sc-271024), IRF3 and phospho-IRF3 (Cell Signaling Cat. No: 11904S and Cat. No: 4947S), STAT1 (Cell Signaling Cat. No: 9172S), phospho-STAT1 (Cell Signaling Cat. No: 9167L), STAT2 (Cell Signaling Cat. No: 72604S), phospho-STAT2 (Cell Signaling Cat. No 4441) and mouse anti-human β-actin (Sigma Cat. No. A2228-200UL), all at 1:1,000 dilution. Secondary antibodies (1:10,000 dilution) were conjugated with IRDye 800 and IRDye 680 (Li-Cor Biosciences), and blots were scanned using an Odyssey infrared imager.

### Cell viability assay

Cell viability was measured in SC at different time points post-transfection and post-infection using the Promega CellTiter 96 AQueous One Solution Cell Proliferation Assay (Cat. No. G3582) as described previously and the absorbance was read at 490 nm on a Victor X Plate Reader (Strange et al., 2019).

### Statistical analysis

ZIKV virus titers, cellular viability data, and gene expression mRNA fold-change are reported as mean ± SEM of at least three independent experiments. GraphPad Prism 5.0 (GraphPad software, San Diego, CA, United States) was used to conduct all statistical analyses. The unpaired Student’s *t*-test was used to compare differences between data from different groups. A value of *p* < 0.05 was considered statistically significant for all analyses.

## Results

### Viral RNA-sensing and type I IFN-associated pathways are functional in human SC

**Figure 1:**
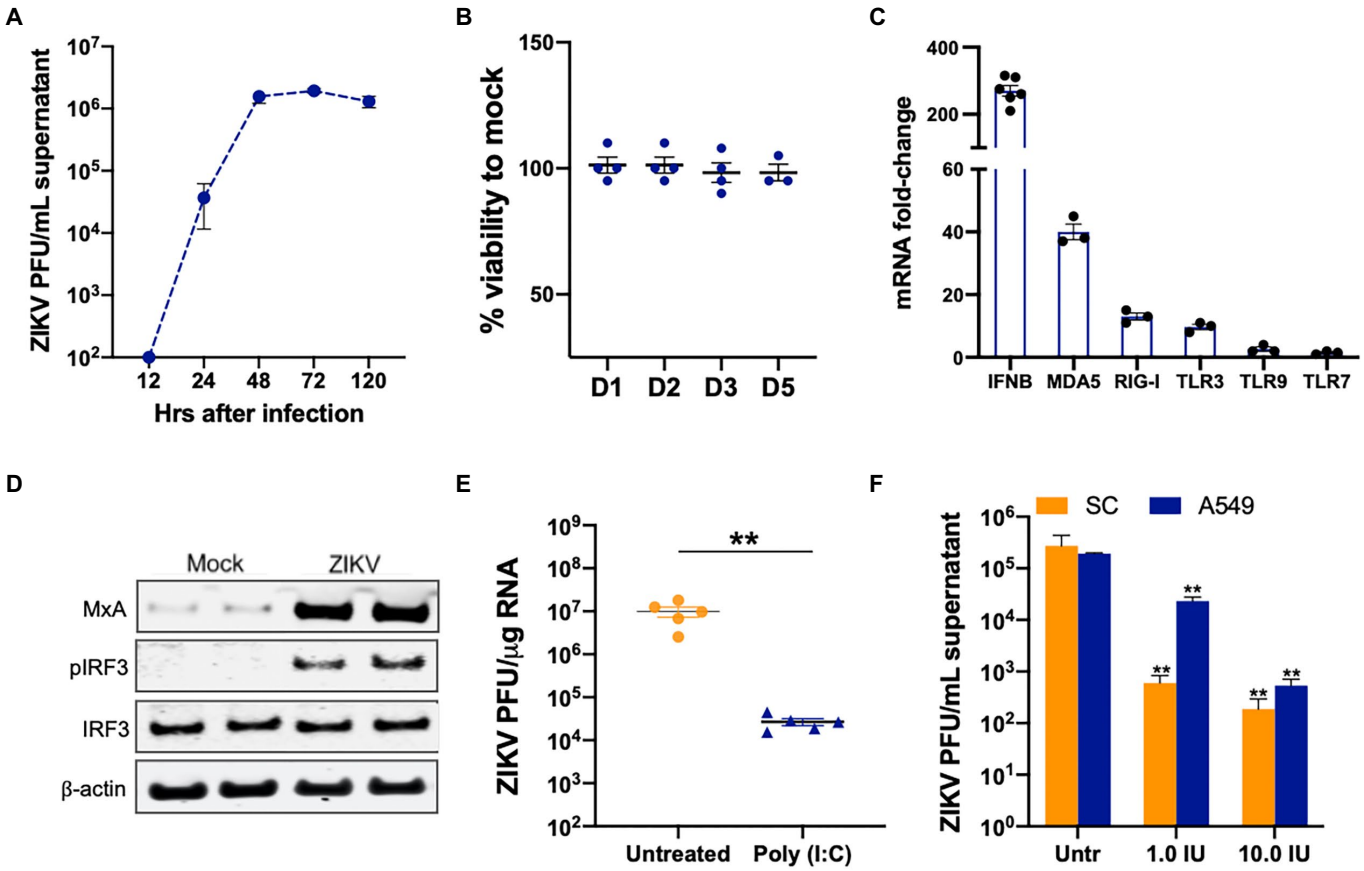
Viral RNA-sensing and type I IFN-associated pathways are functional in SC. **(A)** SC were infected with ZIKV at MOI 1 and ZIKV titers in the supernatant were measured at different time points using plaque assay and expressed as PFU/mL supernatant. **(B)** Cell viability was evaluated in infected SC at different days after infection using CellTiter 96 AQueous One Solution kit and shown as percent cell viability compared to mock-infected cells. **(C)** SC were infected with ZIKV at MOI 1 and gene expression of *IFN-β*, *MDA5*, *RIG-I*, *TLR3*, *TLR7*, and *TLR9* were measured using qRT-PCR at 48 h post-infection (hpi). The fold change was calculated as compared to mock-infected cells and normalized to *GAPDH*. **(D)** Whole-cell extracts from mock and infected SC at 48 hpi were subjected to Western blotting and stained for IRF3, p-IRF3, and MXA. The β-actin was used as a housekeeping loading control and each lane represents an independent experiment. **(E)** SC were pretreated with poly (I:C) 24 h before infection at MOI 1 and ZIKV RNA copies were determined at 48 hpi by qRT-PCR and expressed as ZIKV PFU equivalents per μg RNA. **(F)** SC and A549 cells were pretreated with exogenous IFN-β at two concentrations 24  h before infection and ZIKV titers in the supernatant were measured at 48 hpi using plaque assay and expressed as PFU/mL. Data is presented as mean ± SEM from at least 3 to 5 independent experiments. **p < 0.01 between respective IFN-β treated and untreated cells.

Our previous studies collectively show that ZIKV titers in SC infected with both MOI 1 and 5 reach their peak at 48 h post-infection (hpi) and remain high till day 5 after infection (Siemann et al., 2017; Strange et al., 2019). Further, virus titers correlate well with robust antiviral response suggesting that the antiviral state in neighboring uninfected cells is established only after 48 hpi. Therefore, to evaluate the role of different PRR signaling in controlling ZIKV infection and spread in SCs we used the infectious dose of MOI 1 (Figure 1A) that did not exhibit any cytotoxicity till day 5 after infection (Figure 1B). We first validated our previous RNA Seq data by measuring the changes in the expression of different PRRs by qRT-PCR at 48 hpi. As seen in Figure 1C, ZIKV induced the mRNA expression of PRRs including *MDA5, RIG-I, and TLR3* in SC that correlated with significant induction in *IFN-β*. The induction of *MDA5* was most robust (40-fold), *TLR3* and *RIG-I* were induced between 8 and 12 fold while *TLR 7* and *TLR9* were induced in the range of 2–4 fold. ZIKV-infected SC also showed activation of the transcription factor IRF3, as shown by the increased phosphorylation of IRF3, and increased expression of MXA protein, an indirect marker of IRF3 activation (Figure 1D). We next evaluated whether the downstream signaling of these PRRs is functional in these cells as these PRR pathways are not well characterized in human SC. SC were primed with poly I:C, a dsRNA mimic that is shown to activate TLR3, RIG-I, and MDA5 pathways, 24 h before infection. As expected, poly I:C treatment restricted ZIKV replication by more than 90% (Figure 1E). We further confirmed that the downstream IFN signaling in SC is comparable to other cell types. SC and A549 (lung epithelial cell-derived cell line routinely used to study ZIKV antiviral immunity) were treated with two doses of IFN-β before infection and we observed a significant reduction of ZIKV titers in a dose-dependent manner that was comparable in both cell types (Figure 1F). These data collectively show that viral RNA sensing pathways and IFN signaling are functional and are activated by ZIKV infection in SC.

### Viral RNA-sensing TLRs do not play a role in restricting ZIKV infection in SC

**Figure 2:**
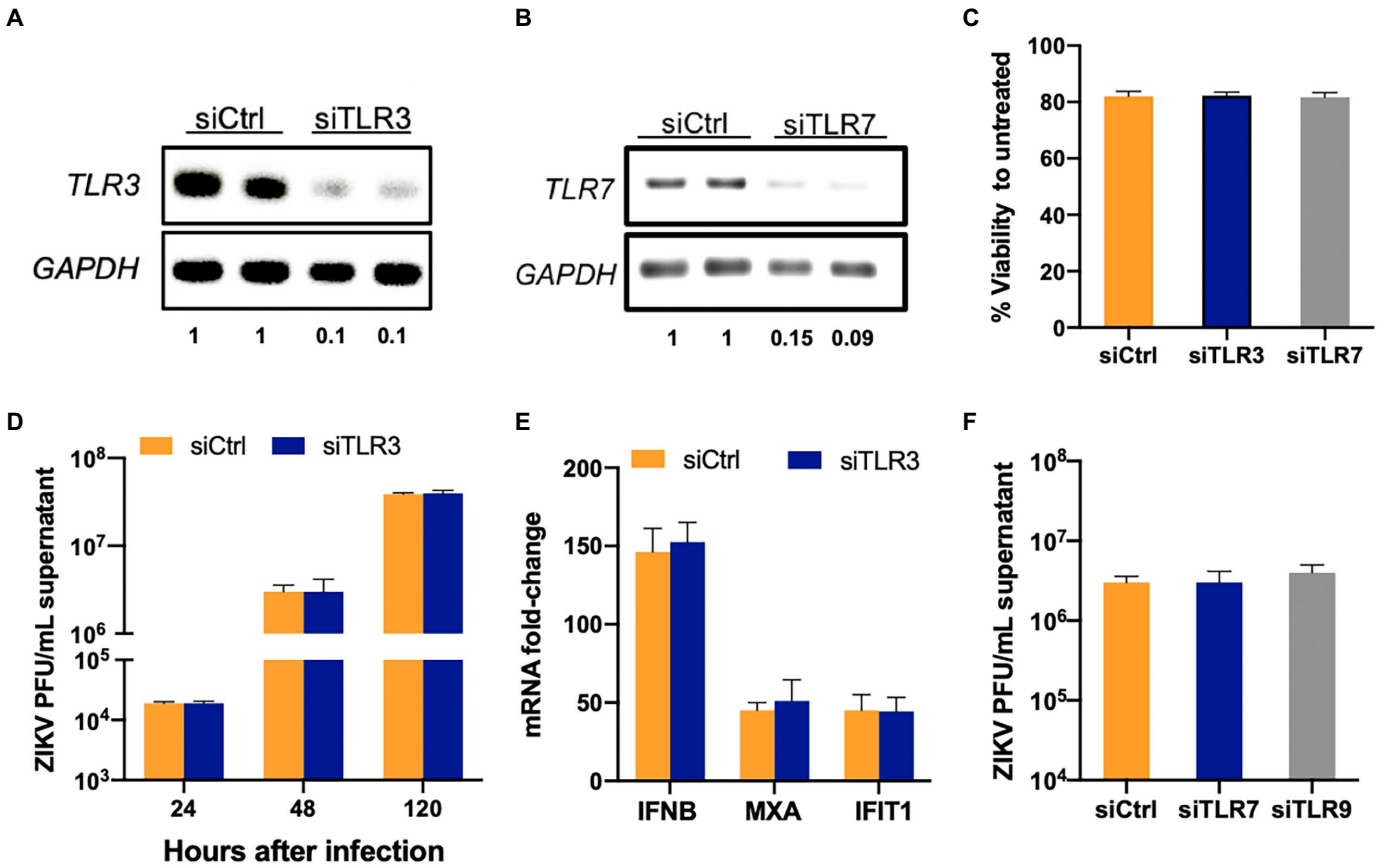
TLR3 and TLR7 are not required to induce an antiviral response in SC following ZIKV infection. SC were transfected with either scrambled control (siCtrl) or **(A)** TLR3 or **(B)** TLR7 specific siRNA and TLR3 and TLR7 expression was measured at 48  h post-transfection (hpt) by RT-PCR to determine silencing efficiency. The values below the bands indicate the ratio of TLR3 or TLR7 mRNA levels in respective silenced cells compared to siCtrl after normalizing to GAPDH **(C)** Cell viability was evaluated in siCtrl, siTLR3-and siTLR7 transfected SC at 48 hpt using CellTiter 96 AQueous One Solution kit and the percent cell viability was calculated as compared to untreated cells. **(D)** TLR3 silenced SC were infected with ZIKV (MOI 1) at 48 hpt and ZIKV titers in the supernatant at different time points were measured by plaque assay. **(E)** Gene expression of *IFNB1, MXA, and IFIT1* was determined at 48 hpi using qRT-PCR. Data was normalized using *GAPDH* and fold change was calculated compared to mock-infected cells. **(F)** ZIKV titers measured by plaque assay in TLR7 and TLR9 silenced SC at 48 hpi. Data is presented as mean ± SEM from at least 3–5 independent experiments.

TLR3 and TLR7 have been shown to sense ZIKV and other flavivirus RNA in various cell types (Hamel et al., 2015; Dang et al., 2016; Ojha et al., 2019). Based on RNA-Seq data that predicted activation of these TLRs, we first tested if they are critical in sensing and controlling ZIKV in SC. TLR3 and TLR7 were silenced in SC using specific siRNA and as seen in Figures 2A,B, the resulting mRNA expression levels of *TLR3* and *TLR7* were reduced by >80% at 48 h post-transfection (hpt) in comparison to controls without significantly compromising cell viability (Figure 2C). The cells were infected at 48 hpt and virus replication was measured. We observed no significant difference in the ZIKV titers in the supernatant at all the time points (24, 48, and 120 hpi) in siTLR3 transfected cells compared to controls (Figure 2D). We further analyzed if the silencing of TLR3 has any effect on the host response parameters. Consistent with the ZIKV replication, inhibition of TLR3 did not significantly affect mRNA expression of the antiviral genes *IFNB1*, *MXA*, and *IFIT1* (Figure 2E). ZIKV titers were also measured in TLR7 silenced SC but no differences were found when compared to siCtrl cells at 24 hpi (data not shown) and 48 hpi (Figure 2F). Similarly, silencing TLR9 using siTLR9 (that reduced gene expression by >70% without affecting the viability, data not shown) also did not change the trend of ZIKV replication (Figure 2F). These results collectively suggest that TLR3, TLR7, and TLR9 do not play an essential role in antiviral response or restricting ZIKV infection in SC.

### RIG-I restricts ZIKV infection in SC

**Figure 3:**
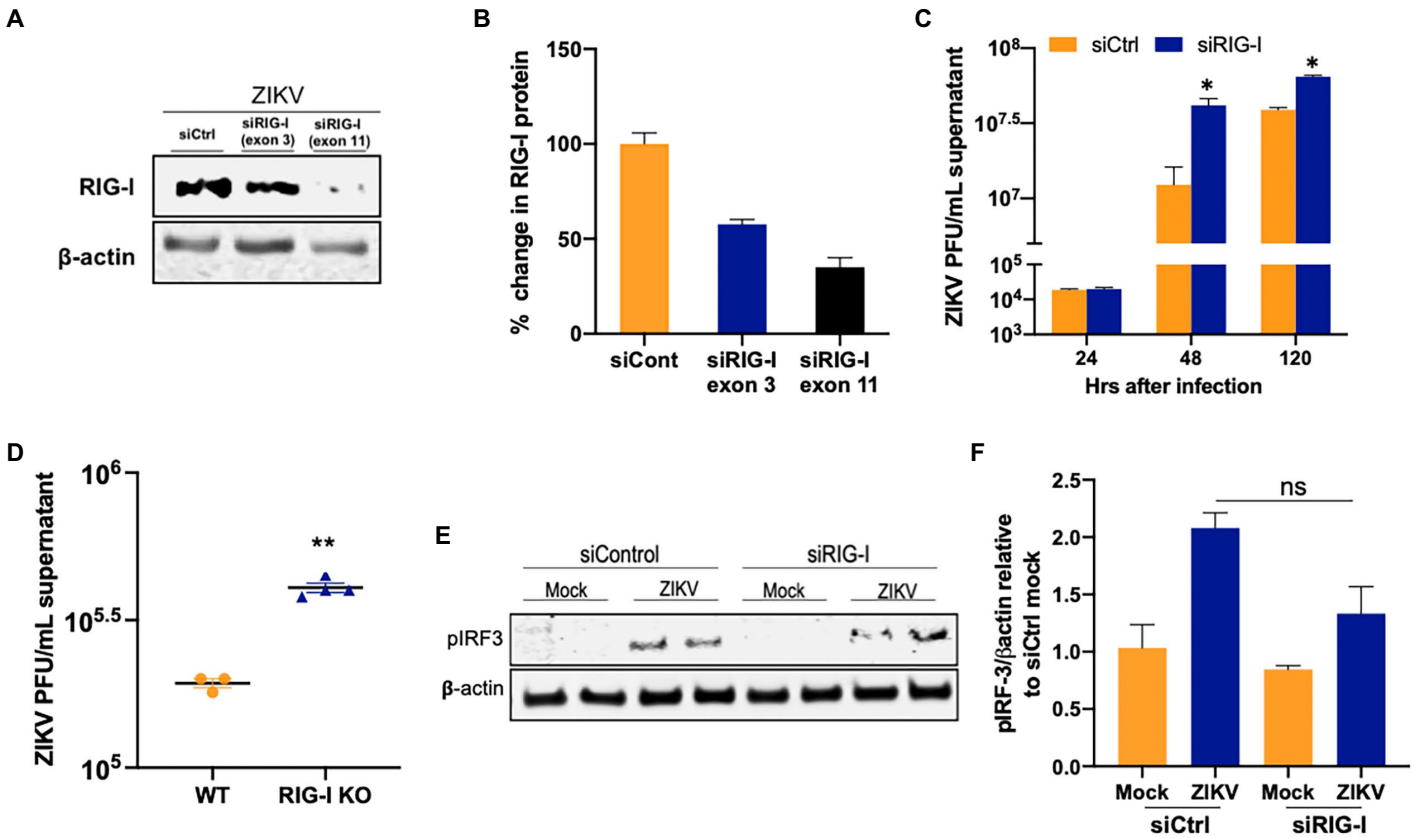
RIG-I restricts ZIKV replication in human SC. **(A,B)** SC were transfected with either siCtrl or siRNA specific to exon 3 or exon 11 of RIG-I. The silencing efficiency was determined by measuring RIG-I protein levels using Western blot at 48 hpt transfection. **(C)** SC were infected with ZIKV (MOI 1) at 48 hpt and ZIKV titers was quantified using plaque assay at different time points. **(D)** Wild-type (WT) and RIG-I knock-out A549 were infected with ZIKV (MOI 1) and virus titers in the supernatants were determined by plaque assay at 48 hpi and expressed as PFU/mL supernatant. **(E)** Whole-cell extracts from uninfected and infected SC transfected with siCtrl, and siRIG-I (exon 11) at 48 hpi with ZIKV were subjected to Western blotting and stained for p-IRF3 and β-actin. **(F)** Densitometry analysis was performed using ImageJ and the p-IRF3/β-actin ratio in infected cells relative to the siCtrl mock was calculated. Data is presented as mean ± SEM from at least 3–5 independent experiments. *p < 0.05; **p < 0.01.

Based on the well-documented role of RIG-I in restricting ZIKV infection in other cell types, we next sought to determine whether RIG-I signaling plays a similar role in SC. We used two siRNAs targeting exon 3 and exon 11 of *DDX58* to block RIG-I mRNA expression. Subsequent Western blotting analyses showed that the silencing efficiency at the protein level was 50 and 70% for exon 3 and 11 siRNAs, respectively (Figures 3A,B), with no significant change in viability at 48 hpt (data not shown). The difference at 24 hpi was not significant between the two groups, however, in contrast to TLRs, ZIKV titers increased in SC treated with siRIG-I exon 11 by almost 350% as compared to siCtrl treated cells at 48 hpi and the trend was also maintained at 120 hpi (Figure 3C). Since RIG-I is shown to control ZIKV replication in A459 cells, a lung epithelial cell line, routinely used to study ZIKV antiviral immunity (Lin et al., 2019), we also compared our data with A549 cells deficient in RIG-I. The percent increase in virus titers in SC was comparable to the increase in titers in A549 at 48 hpi (Figure 3D). We further compared the protein expression of the phosphorylated IRF3 (p-IRF3) using western blot, and as expected there was a marked increase in the p-IRF3 in siControl treated cells at 48hpi (Figures 3E,F). However, densitometric scanning analysis showed that although there was a trend of decrease in the p-IRF3 protein levels in SC silenced with siRIG-I, it was not statistically significant suggesting that silencing RIG-I has only a moderate effect in attenuating downstream signaling in these cells. These results collectively suggest that RIG-I is one of the PRRs involved in controlling ZIKV infection in SC.

### MDA5 restricts ZIKV infection in SC

**Figure 4:**
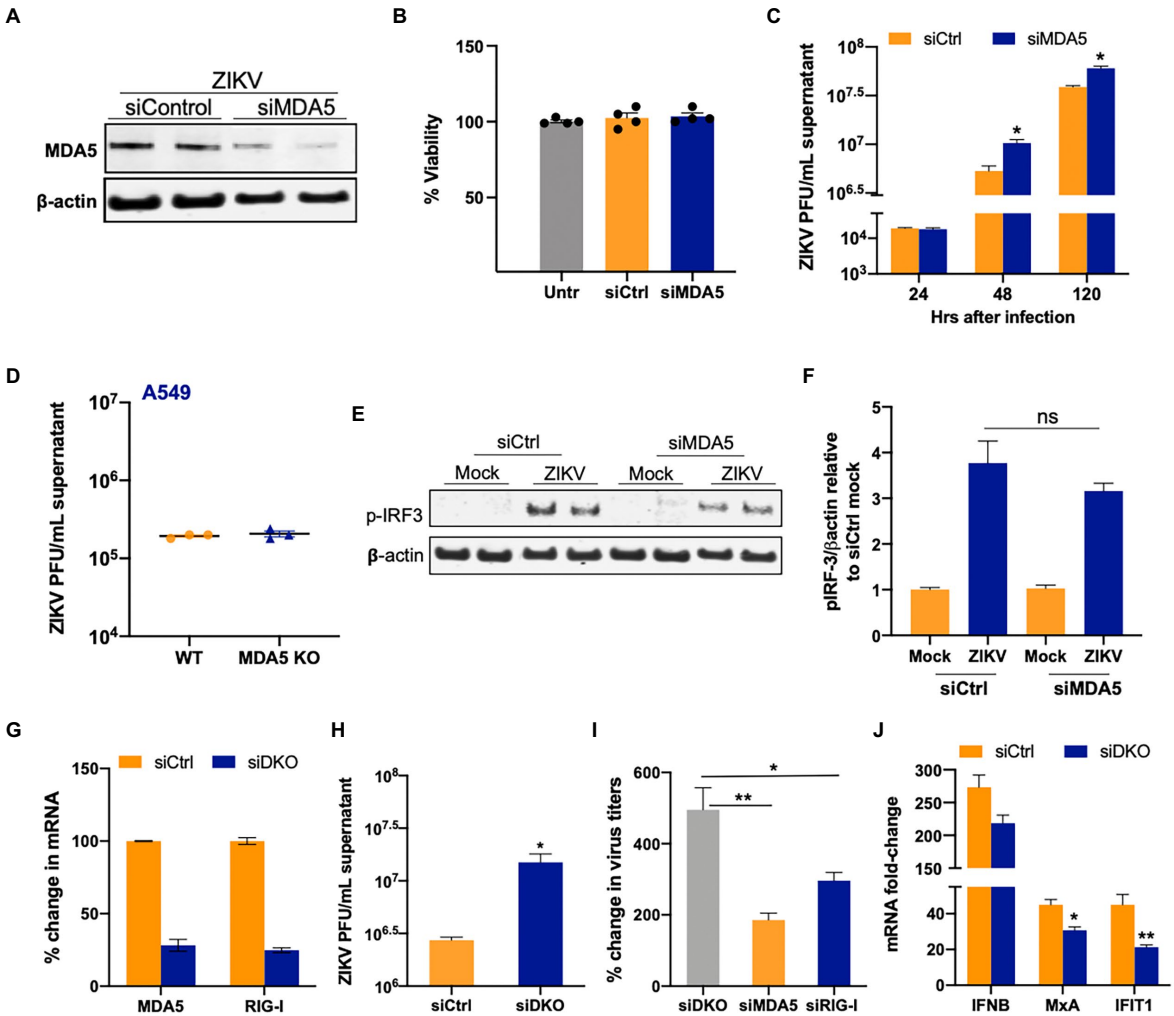
Silencing of MDA5 increases ZIKV replication in SC. **(A)** SC were transfected with siRNA specific for siMDA5 or siCtrl and silencing efficiency was determined by measuring MDA5 protein levels using Western blot at 48 hpt. **(B)** Percent change in the cell viability of siCtrl and siMDA5 transfected SC at 48 hpt was calculated as compared to untreated cells. **(C)** SC were infected with ZIKV (MOI 1) at 48 hpt and ZIKV PFU were determined by plaque assay at different time points, expressed as PFU/mL supernatant. **(D)** WT and MDA5 knockout A549 were infected with ZIKV (MOI 1) and virus titers in the supernatants were determined by plaque assay at 48 hpi. **(E)** The effect of siMDA5 on phosphorylated IRF3 (p-IRF3) protein expression was determined in both mock-and ZIKV-infected SC at 48 hpi by Western blotting. **(F)** Densitometry analysis was performed to quantify western blot data and expressed as the ratio of p-IRF3/β-actin in infected cells relative to the siCtrl mock. **(G)** SC were co-transfected with siMDA5, and siRIG-1 (exon 11), and knockdown was determined by measuring mRNA transcripts. **(H)** ZIKV titers in the supernatants from siCtrl and double knock-down (DKD) with siMDA5 and siRIG-I cells were determined by plaque assay at 48 hpi and expressed as PFU/mL supernatant. **(I)** Percent increase in ZIKV titers in SC transfected with siRIG-I and siMDA5 compared to siCtrl at 48 hpi. **(J)** Gene expression of *IFNB1, MXA, and IFIT1* was determined at 48 hpi using qRT-PCR. Data was normalized using GAPDH and fold change was calculated compared to respective mock-infected cells. Data represent mean ± SEM from at least 3–5 independent experiments. *p < 0.05; **p < 0.01.

Since RIG-I and MDA5 have nonredundant roles in detecting flavivirus infection (Errett et al., 2013) and because *MDA5* induction was most robust in SC, we next tested the ability of MDA5 signaling to restrict ZIKV infection in SC. To attenuate the MDA5 pathway in SC, we treated the cells with siMDA5 and measured MDA5 protein expression at 48 hpt. As shown in Figure 4A, the expression of MDA5 was reduced by 60–70% at the protein level based on densitometric scanning analysis and the transfection did not compromise the cell viability (Figure 4B). To determine the effect of MDA5 silencing on ZIKV replication, SC were infected with ZIKV (MOI 1) at 48 hpt and virus titers were subsequently measured at 24, 48, and 120 hpi. Similar to what we observed with RIG-I silencing, MDA5 silencing also led to a significant increase (~200%) in ZIKV titers as compared to siCtrl cells only at 48 and 120 hpi (Figure 4C). We also compared the effect of MDA5 knockout on ZIKV replication in A549 cells. Interestingly, we observed that, unlike SC, loss of MDA5 in A549 did not lead to increased virus titers (Figure 4D), which was in agreement with other studies (Schilling et al., 2020) thus again supporting the cell-type specific role of different PRRs in virus-control. We further validated the association between silenced MDA5 and antiviral response and observed that there was a slight reduction in p-IRF3 levels in MDA5-silenced SC compared to control at 48 hpi (Figures 4E,F), however, it was not statistically significant. To further understand the synergy between the two RLRs, ZIKV titers were also determined in SC silenced with both RIG-I and MDA5 (Figure 4G). ZIKV titers in the double knockdown SC were significantly higher than the siCtrl cells at 48 hpi (Figure 4H). The percent increase of ZIKV titers in SC co-transfected with siRIG-I and siMDA5 was also significantly greater than the percent increase observed in individually silenced SCs (Figure 4I). Further, increased virus titers in the double knockdown cells correlated with decreased IFN response including mRNA expression of *IFIT1* and *MxA* at the same time point (Figure 4J). These results collectively indicate that MDA5 is also one of the key PRRs in the response and restriction of ZIKV infection in SC, operating in parallel with RIG-I.

### TGF-β1 and Activin A have no effect on ZIKV infection in SC

**Figure 5:**
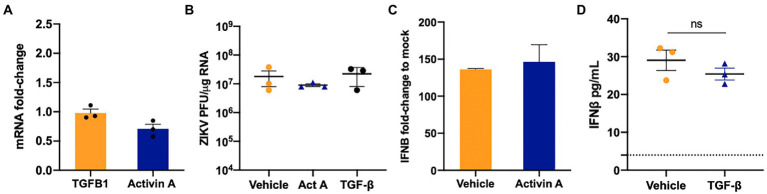
TGF-β1 and Activin do not regulate ZIKV replication in SC. **(A)** Gene Expression of *TGFB1* and *INHBA* (Activin A) was measured at 48 hpi using qRT-PCR and fold change was calculated as compared to mock-infected cells after normalizing to *GAPDH*. **(B)** SC were treated with 15  ng/mL of human recombinant TGF-β1 protein or 100  ng/mL Activin 24  h before ZIKV infection at MOI 1 and ZIKV PFU equivalents per microgram of RNA were determined at 48 hpi by qRT-PCR. **(C)** Gene expression of *IFNB1* was determined in Activin A exposed SC at 48 hpi using qRT-PCR. **(D)** Secreted IFN-β protein levels in SC supernatant at 48 hpi was determined by ELISA; the dotted line indicates the minimal detection limit of the assay. Data is presented as mean ± SEM from at least 3–5 independent experiments.

Our data in Figures 1–4 collectively show that although ZIKV is sensed by MDA5 and RIG-I and downstream antiviral signaling is activated, they were not capable of completely restricting ZIKV replication. Furthermore, we and others have shown that immunosuppressive pathways active in SC like Axl may modulate anti-ZIKV response (Siemann et al., 2017; Kumar et al., 2018). Therefore, we next evaluated if ZIKV infection alters the mRNA expression of TGF-β superfamily members, TGF-β1 and Activin A. As seen in Figure 5A, ZIKV does not induce changes in the mRNA expression of *INHBA* (Activin A) or *TGF-β1*. Since these molecules are also produced in high amounts by other testicular resident cells like LC, we next evaluated if exogenous exposure of TGF-β1 (10 and 15 ng/mL) or Activin A (50 and 100 ngmL) can affect ZIKV replication in SC. We found that exogenous treatment with recombinant TGF-β1 or Activin A at both lower (data not shown) and higher (Figure 5B) concentrations showed no significant change in ZIKV RNA. We further confirmed that the exposure of SC to these molecules also did not affect IFN-β levels (Figures 5C,D). The data suggest that the two TGF-β superfamily members TGF-β1 and Activin A do not respond to ZIKV infection of SC and do not play a role in modulating ZIKV replication and antiviral immunity.

### BMP6 modulates ZIKV replication and regulates the expression of key ISGs in SC

**Figure 6:**
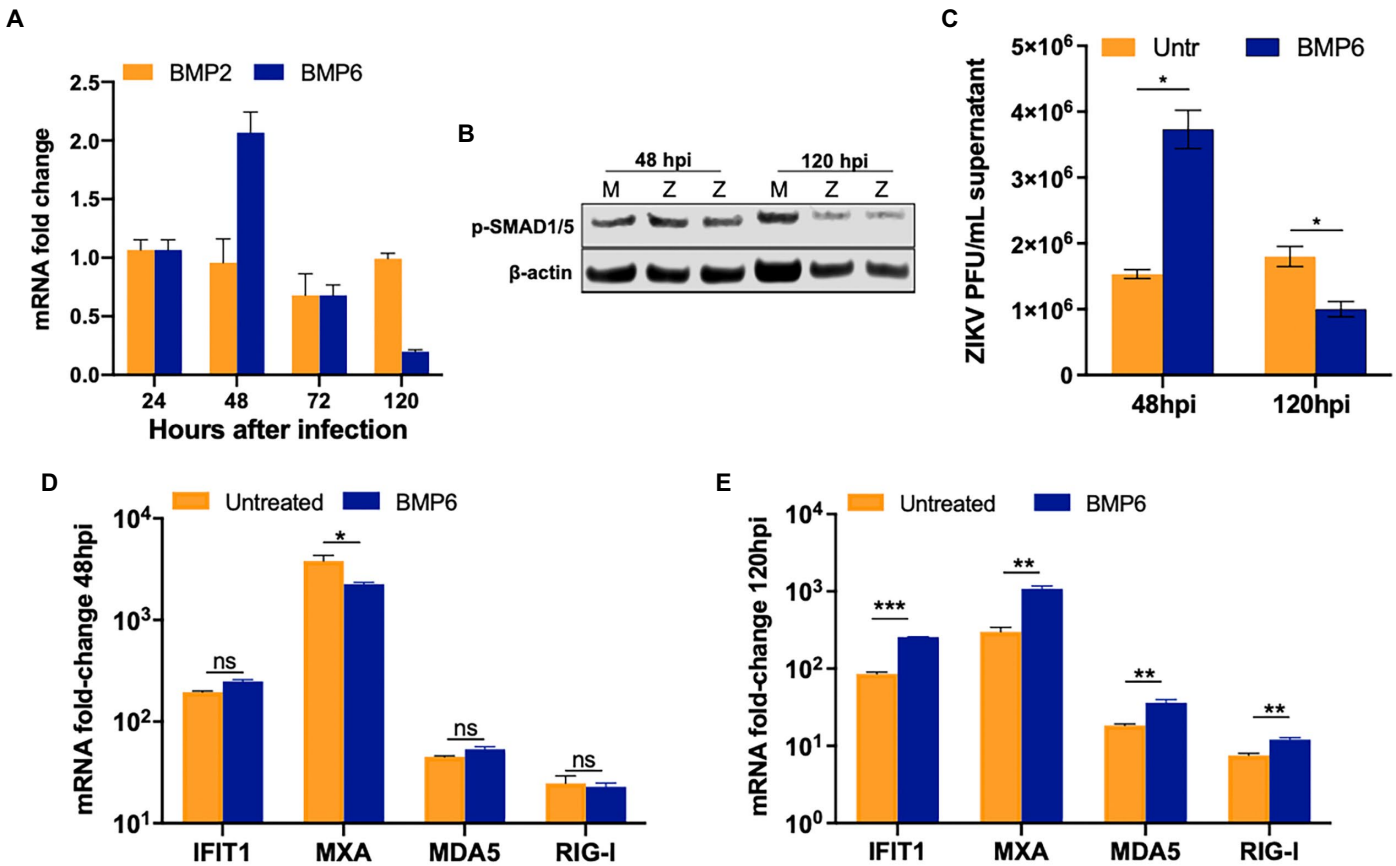
ZIKV replication impairs BMP6 signaling in SC. **(A)** BMP2 and BMP6 mRNA fold-change measured in SC infected with ZIKV at MOI 1. **(B)** Western blot analysis of phosphorylated SMAD1/5 (pSMAD1/5) at different time points after infection, β-actin was used as housekeeping loading control; each lane represents an independent experiment. **(C)** SC were pretreated with 100  ng/mL exogenous human recombinant BMP6 24  h before ZIKV infection at MOI 1, replenished every 24  h, and virus titers in the supernatant were assayed by plaque assay at 48 hpi. Gene expression of *IFIT1, MXA, MDA5*, and *RIG-I* were measured using qRT-PCR at **(D)** 48 hpi and **(E)** 120 hpi, and fold change was calculated as compared to mock-infected cells after normalizing to *GAPDH*. Data is presented as mean ± SEM from at least 3–5 independent experiments. *p < 0.05; **p < 0.01; ***p < 0.001.

BMP6, another member of the TGF-β superfamily, and its receptors are highly expressed in SC and regulate cell proliferation (Wang et al., 2017, p. 6; Ni et al., 2019). Further, ZIKV has been recently shown to induce BMP2 signaling in human brain pericytes (Chen et al., 2021). Therefore, we next assessed the gene expression of two key BMPs (BMP2 and BMP6) at different time points after infection. While ZIKV replication did not alter the levels of BMP2, we observed that BMP6 was modestly upregulated at 48 hpi by almost two-fold; however, by 120 hpi, BMP6 expression decreased significantly by >70% (Figure 6A). Additionally, the expression of the phosphorylated SMAD1/5, a marker of activated BMP6 signaling, decreased by 25–30% at 120 hpi as compared to mock controls, thus following the same trend of BMP6 mRNA expression (Figure 6B). Next, to assess whether BMP6 affects ZIKV replication, SC were treated with BMP6 at the concentration of 60 and 100 ng/mL based on other studies (Wang et al., 2017) for 24 h before infection and then replenished with fresh BMP6 every 24 h. We found that only 100 ng/mL BMP6 treatment resulted in an increase in viral titers at 48 hpi and a decrease by 50% at 120 hpi as compared to untreated SC (Figure 6C). We further examined the effect of BMP6 on the antiviral response and found that while the expression of key ISGs did not change drastically at 48 hpi (Figure 6D), the mRNA levels of *MXA* and *IFIT1* increased significantly in BMP6 treated SC at 120 hpi by almost 3–4 fold (Figure 6E). Similarly, BMP6 treatment also induced mRNA expression of *RIG-I* and *MDA5* at higher levels (~70%, Figure 6E) that correlated with decreased virus titers observed in these cells (Figure 6C). Together, these data suggest that BMP6 signaling is suppressed in SC at later stages of ZIKV infection and is one of the modulators of anti-ZIKV response in this cell type.

### BMP6 potentiates the antiviral response in SC

**Figure 7:**
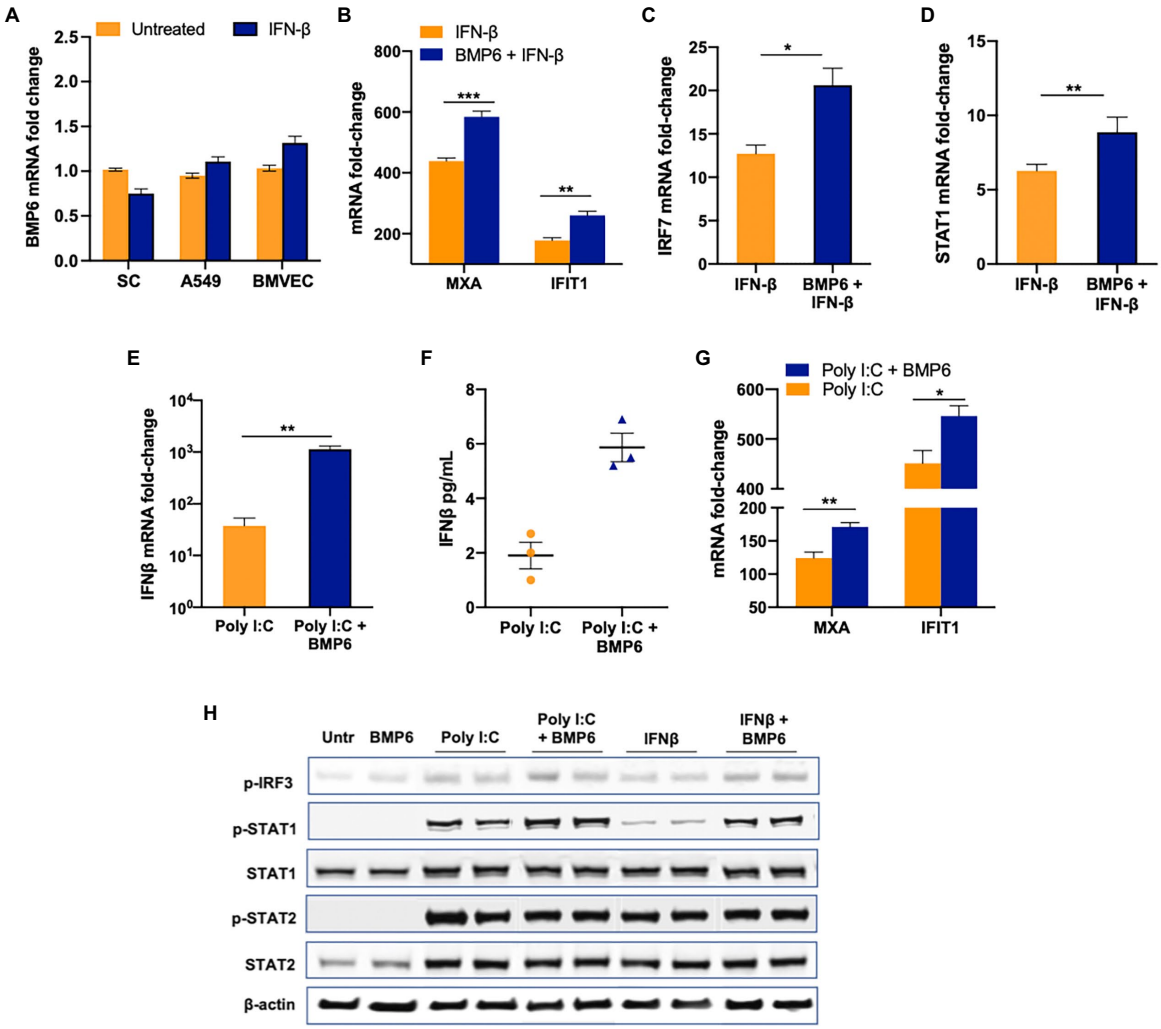
BMP6 enhances the antiviral response in SC. **(A)** BMP6 mRNA transcripts were measured in SC, A549, and BMVEC at 24  h post-treatment with 1  IU/mL of recombinant human IFN-β using RT-PCR. **(B–D)** SC were treated with recombinant human IFN-β in the presence and absence of recombinant BMP6 (100  ng/mL) for 24  h and mRNA transcripts of *MXA, IFIT1, IRF7*, and *STAT1* were measured by qRT-PCR. SC treated with poly (I:C) in the presence and absence of BMP6 and mRNA expression of **(E)** *IFNB1*, and **(G)** *IFIT1, and MXA* was measured by qRT-PCR. **(F)** IFN-β levels in the supernatant from treated SC were measured by ELISA and expressed as pg/mL supernatant. **(H)** Whole-cell extracts from IFN-β and poly I:C exposed SC in the presence and absence of BMP6 were subjected to Western blotting and stained for p-IRF3, total and p-STAT1, total and p-STAT2, and β-actin. Data is presented as mean ± SEM from at least 3–5 independent experiments. *p < 0.05; **p < 0.01; ***p < 0.001.

The increase in ISGs expression in BMP6-treated SC suggested that there might be crosstalk between the innate immune pathways and BMP6 signaling. To further validate the association between the antiviral response against ZIKV infection and BMP6 signaling, we first tested if BMP6 expression is under the control of IFN-β in SC and two other cell types (A549 and BMVEC). Our result showed no significant change in the expression of BMP6 in all three cell types at 24 h post-treatment of 1 IU/mL of IFN-β suggesting that BMP6 expression is not regulated by IFN-β (Figure 7A). Similarly, poly I:C treatment also did not affect BMP6 expression at 24 h post-treatment (data not shown) suggesting that BMP6 expression is not directly regulated by both molecules. We next examined whether BMP6 in turn can modulate IFN-β-associated antiviral response by treating SC with both BMP6 and IFN-β. Interestingly, treatment of BMP6 (100 ng/mL) increased the expression of ISGs including *IFIT1*, *MXA*, *IRF7*, and *STAT1* (Figures 7B–D). To further evaluate BMP6 crosstalk with the antiviral response, we next treated SC with poly I:C in the presence and absence of BMP6. As seen in Figures 7E,F, BMP6 treated group exhibited significantly increased *IFNB1* transcription compared to poly I:C treatment alone which also led to increased IFN-β levels in the supernatant media from cells at 24 h after treatment with poly I:C and BMP6 (Figure 7F). The increase in IFN-β correlated with a significant increase in the levels of *IFIT1* and *MXA* transcripts (Figure 7G) in SC exposed to both poly I:C and BMP6. However, BMP6 treatment alone in the absence of poly I:C or IFN-β did not affect the basal expression of these antiviral genes compared to untreated controls (data not shown). To further assess the involvement of downstream transcription factors in BMP6 crosstalk, we measured the levels of p-IRF3, a critical transcription factor in RLR signaling, in SC after 24 h of treatment with IFN-β and poly I:C in the presence and absence of BMP6. While the levels of p-IRF3 were very low and not different between the untreated and BMP6 exposed cells, the levels of p-IRF3 markedly increased in both IFN-β and poly I:C treated cells. However, the presence of BMP6 further increased p-IRF3 levels in both poly I:C and IFN-β treated cells by 40 and 60%, respectively, (Figure 7H). Analysis of transcription factors involved in IFN signaling also demonstrated a significant increase in p-STAT1 levels in SC treated with both BMP6 and poly I:C and IFN-β by 100 and 350%, respectively. However, there was no difference between p-STAT2 levels in BMP6 treated and untreated groups (Figure 7H). Overall, these data showed that although type I IFN does not directly affect the *BMP6* expression, BMP6 signaling is involved in positively regulating antiviral response in SC.

## Discussion

SC are responsible for creating an immunosuppressive environment in the testes by secreting molecules that promote immune tolerance; however, SC are also the primary responders to many testicular viral infections (Zhao et al., 2014). Our previous transcriptomics data showed that ZIKV can trigger an innate immune response through predicted activation of multiple PRR pathways in SC (Strange et al., 2018a) leading to other important questions as to which virus sensing pathways play an active role in controlling ZIKV replication in SC and whether immunosuppressive pathways in SC dampen this effect. Here, we elucidate the impact of silencing multiple PRR pathways on ZIKV replication in SC. The highlights of our study are (i) canonical viral RNA-sensing is functional in SC; however, (ii) TLR3, 7 and 9 do not affect ZIKV replication outcome, whereas RIG-I and MDA5 play a dominant role in inducing an anti-ZIKV response; (iii) ZIKV does not modulate SC-specific immunosuppressive pathways including TGF-β and Activin A; however, BMP6 signaling is suppressed at later stages of infection; and (iv) BMP6 positively regulates antiviral response, downregulation of which may likely affect virus clearance and thus suggests a novel crosstalk mechanism between antiviral response and the BMP6 pathway in SC.

The testis is an immune privileged organ wherein the systemic immune responses to autoantigens and alloantigens are attenuated, however broad spectrum of pathogens can infect testicular cells. Although the role of SC in the activation of innate immune defense against bacterial infections is well characterized, our understanding of virus-sensing mechanisms is limited in human SC, as most studies have used murine SC. It is shown that mouse SC expresses multiple TLRs and RLRs and can activate robust inflammatory response in response to viruses like mumps virus (MuV) (Wu et al., 2016). Similarly, a recent *in vivo* study (Qin et al., 2019) reported that exposure to poly I:C induced IFN and inflammatory response *via* TLR3 and impaired blood-testis barrier in mouse testes. However, the underlying mechanisms of innate immune activation in human testicular cells are yet to be intensively investigated. Our data in Figure 1 provides evidence that human SC can induce an antiviral state when primed with poly I:C, a viral-like PAMP that can effectively restrict ZIKV infection by >90%. This response in SC follows the same trend as seen in other human cell types such as Huh-7 cells, where poly I:C treatment induced IFN-β and ISGs, and attenuated ZIKV infection (Tricot et al., 2018). Similarly, IFN-β treatment has been shown to suppress ZIKV in many cell types (Lin et al., 2019; Gobillot et al., 2020; Strange et al., 2021). Collectively, this suggests that the persistence of ZIKV in SC is not likely due to a significant defect in virus sensing or IFN signaling pathways (Bowen et al., 2017; Caine et al., 2019; Lin et al., 2019).

Because our previous study predicted activation of multiple PRR signaling pathways (Strange et al., 2018a), we examined the role of multiple PRRs in inducing downstream anti-ZIKV responses to understand underlying mechanisms. Previous ZIKV studies have shown the upregulation of TLR3 in various cell types including human primary retinal pigment epithelial cells, astrocytes, skin fibroblast, and mouse neurospheres (Hamel et al., 2015; Dang et al., 2016; Singh et al., 2017; Ojha et al., 2019). Further, TLR3 activation was found to be associated with the depletion of neural progenitor cells in ZIKV-infected human cerebral organoids (Dang et al., 2016). Similarly, data from human myeloid cells showed that cells treated with TLR7 agonist R848 block ZIKV replication through inductions of ISGs (Vanwalscappel et al., 2018). However, our data does not follow this trend and shows that TLR3 and TLR7 are not involved in inducing innate immune responses to ZIKV in SC. The underlying reasons are not clear but as suggested by the studies involving mumps virus in mouse SC (Wu et al., 2016), we speculate that TLR3/7 pathways may not be functional in human SC.

The main highlight of our data is that ZIKV induced innate immune responses through both RIG-I and MDA5 signaling in SC. RIG-I and MDA5 play both redundant and nonredundant functions in controlling many flaviviruses including WNV, DENV, and ZIKV, despite signaling through the shared adaptor protein, MAVS (Wilkins and Gale, 2010; Loo and Gale, 2011; Olagnier et al., 2014; Serman and Gack, 2019; Stone et al., 2019). ZIKV infection induced expression of RIG-I and MDA5 mRNA in human skin fibroblasts and DC (Hamel et al., 2015; Bowen et al., 2017); however, only silencing of RIG-I significantly increased virus replication in fibroblasts (Hamel et al., 2015). Similarly, RIG-I was shown to be the main sensor of ZIKV in A549 cells (Schilling et al., 2020). However, studies investigating human trophoblasts showed the ablation of both RIG-I and MDA5 significantly increased ZIKV infection (Ma et al., 2018) suggesting a cell type-specific role of these pathways. The virus sensing pathways by cytoplasmic sensors like RIG-I and MDA5 are not well studied in immunosuppressive SC, therefore our data may have implications in understanding testicular innate antiviral immunity to other RNA viruses as well. Interestingly, although silencing of both RIG-I and MDA5 increased ZIKV replication in SC, the increase in virus titers was not as robust as seen in other cell types such as trophoblast cells and A549, where RIG-I silencing increased ZIKV titers by >1 log. We believe that there might be two possible explanations for this. First, virus proteins are able to more efficiently antagonize antiviral response in SC compared to other cell types (Serman and Gack, 2019) and that may facilitate continued replication. The second explanation is that other immunoregulatory molecules enriched in SC may affect the efficiency of PRR-induced responses.

The key immune molecules/pathways critical to maintaining an immunosuppressive environment in the testis include TAM receptor family, TGF-β superfamily, MAPK, and AMPK (Ni et al., 2019). We recently demonstrated that Axl, one of the TAM receptors, promotes ZIKV entry and negatively regulates the antiviral state of SC to augment ZIKV infection (Strange et al., 2019). AMPK pathway has been recently shown to exhibit anti-ZIKV effects in human endothelial cells (Singh et al., 2017). The TGF-β superfamily includes activin, inhibin, BMPs, and TGF-β homodimeric proteins, which are all highly expressed in the testis including SC, and collectively regulate many functions of SC such as proliferation, differentiation, and apoptosis (Ni et al., 2019). Accumulating studies implicate these TGF-β members also respond to virus infections including SARS-CoV-2 and HCV (Chusri et al., 2016; Ferreira-Gomes et al., 2021). High levels of TGF-β expression were reported in HCV patients, as well as in HCV-infected A549 cells (Mirzaei and Faghihloo, 2018). In ZIKV-infected mothers who delivered infants with microcephaly, high levels of TGF-β were found in the plasma. Furthermore, Eddowes and colleagues showed Activin A treatment suppressed the growth of ZIKV in the lung epiethelial cells derived cell line A549 (Eddowes et al., 2019). However, our data clearly shows that ZIKV does not modulate either TGF-β1 or Activin A expression, and exogenous treatment of TGF-β and Activin A also does not affect ZIKV replication kinetics. These data suggest that unlike shown in other cell types, these molecules do not have any direct or indirect effect on anti-ZIKV response in SC.

The BMP family of proteins has generated a lot of interest with respect to their ability to modulate different biological functions in addition to bone formation in a cell-specific manner. However, the effect of BMPs on the immune system is relatively less studied in comparison to TGF-β. Some of the well-documented roles of BMP in the immune system include the skewing of macrophage differentiation into M2 phenotype by BMP7 and 4 (Sconocchia and Sconocchia, 2021). Other BMPs like BMP2 are shown to induce cell adhesion molecules and promote chemotaxis of monocytes thus promoting inflammation in endothelial cells (Pardali et al., 2018). The BMP6 can also signal through a non-canonical pathway that involves PI3K-AKT kinases, TRAFs, and JNK/ERK MAP kinases. While the antiviral activity of BMP6 has not been explored in ZIKV and other flaviviruses, a recent study reported activation of BMP2 maturation in ZIKV-infected brain pericytes that led to osteogenic gene expression and calcification (Chen et al., 2021). ZIKV suppressed BMP6 expression as well as the downstream signaling as evidenced by decreased phosphorylation of SMAD1/5 at the later stages of infection. These results also prompted us to evaluate if BMP6 is under the control of IFN. However, the observation that IFN-β does not alter the expression of BMP6 in SC, A549, and HBMVEC strongly suggests that BMP6 expression is not regulated by type I IFNs. On the other hand, our results in Figure 7 are interesting and provide strong evidence that BMP6 potentiates the production of IFNB1 transcripts and downstream response as seen by increased expression of *MXA* and *IFIT1*. Similarly, the further increase in the p-IRF3 and p-STAT1 levels in BMP6-treated cells support the notion that BMP6 potentiates antiviral response. Similarly, BMP6 and Activin A were shown to suppress the growth of HBV in cell culture (Eddowes et al., 2019). This study also showed that BMP6 treatment increased IFN activity, especially *MDA5* expression in Huh.7 cell line. The underlying mechanism by which BMP6 facilitates IFN signaling is not clearly characterized as of yet, however, based on the data demonstrating the interaction between SMADs and IRF7 (Qing et al., 2004), it is likely that BMP6 affects the transcription factors involved in IFN signaling (IRF3 and 7) *via* SMAD5/6. Our recent study has shown the link between RLR signaling and SMAD4 in West Nile virus-infected macrophages (Stone et al., 2019) suggesting crosstalk between SMADs and RLR signaling in flaviviruses. Based on our collective data, we believe that the suppression of BMP6 by ZIKV at later stages may have an implication for continued virus replication in SC. It is likely that BMP6 suppression during the later stages of infection may be a virus-induced response to negatively impact the robustness of the interferon response, possibly another mechanism by which ZIKV antagonizes the IFN response. However, in this study, we only analyzed a limited repertoire of genes regulated by BMP6 in SC and our future studies will focus on further understanding how canonical and non-canonical pathways of BMP6 interconnect with IFN and antiviral response in SC and other cell types. In summary, our data indicate that the innate immune response to ZIKV in SC is induced through RIG-I and MDA5 pathways, and modulation of BMP6 and downstream signaling by ZIKV at later stages of infection may negatively modulate antiviral response and facilitate virus persistence in infected SC. These results fill gaps in our understanding of the antiviral signaling mechanisms underlying ZIKV infection and persistence in human testes.

## Data availability statement

The raw data supporting the conclusions of this article will be made available by the authors, without undue reservation.

## Author contributions

SV, BJ, DS, and MG conceptualized the study. BJ, NP, DS, and SG conducted the experiments. BJ, DS, SG, NP, and SV analyzed the data. BJ and SV wrote the first draft, and others were involved in editing. MG provided resources. SV provided funds for this study. All authors contributed to the article and approved the submitted version.

## Funding

This work was partially supported by grants R21AI129465 (SV), R21AI140248 (SV), Hawaii Community Foundation (SV), R01AI145296 (MG) and R01AI143265 (MG). BJ was supported by an NIH Minority Health Research Training program grant (T37MD008636-08).

## Conflict of interest

The authors declare that the research was conducted in the absence of any commercial or financial relationships that could be construed as a potential conflict of interest.

## Publisher’s note

All claims expressed in this article are solely those of the authors and do not necessarily represent those of their affiliated organizations, or those of the publisher, the editors and the reviewers. Any product that may be evaluated in this article, or claim that may be made by its manufacturer, is not guaranteed or endorsed by the publisher.
